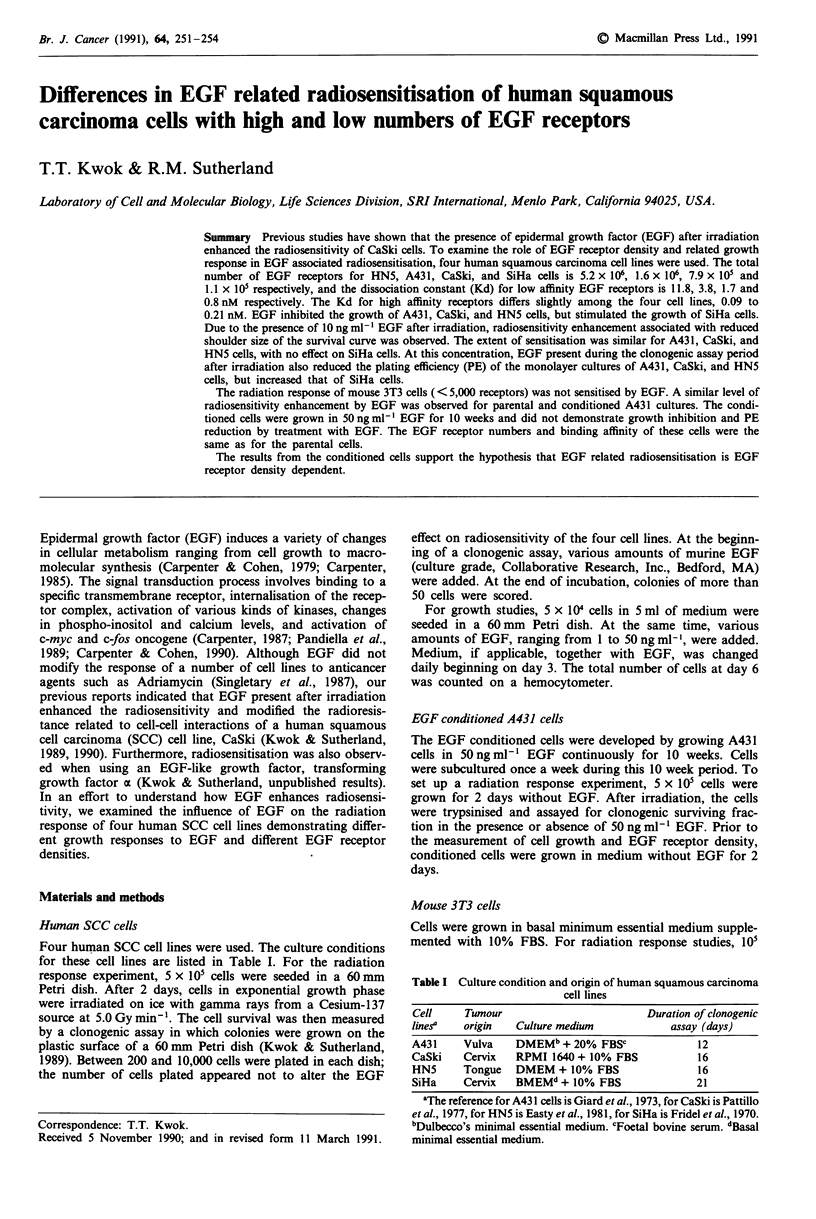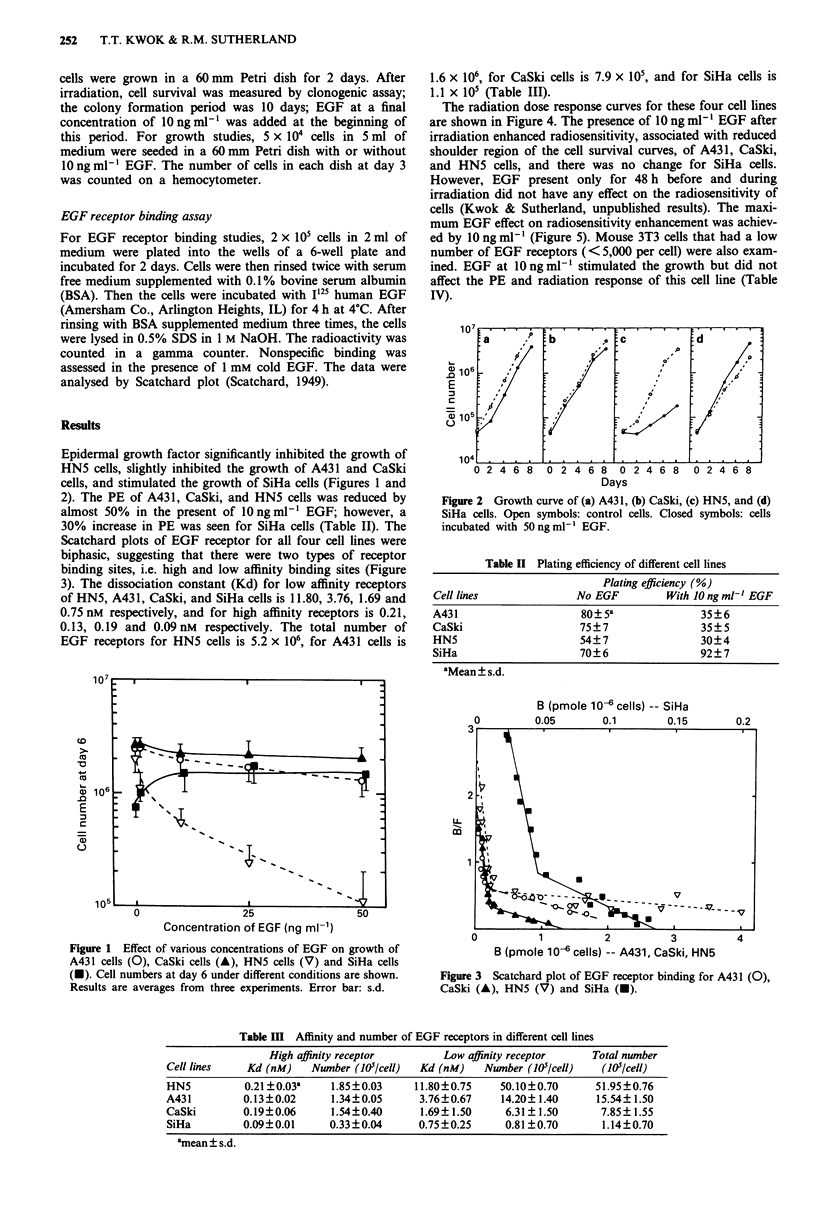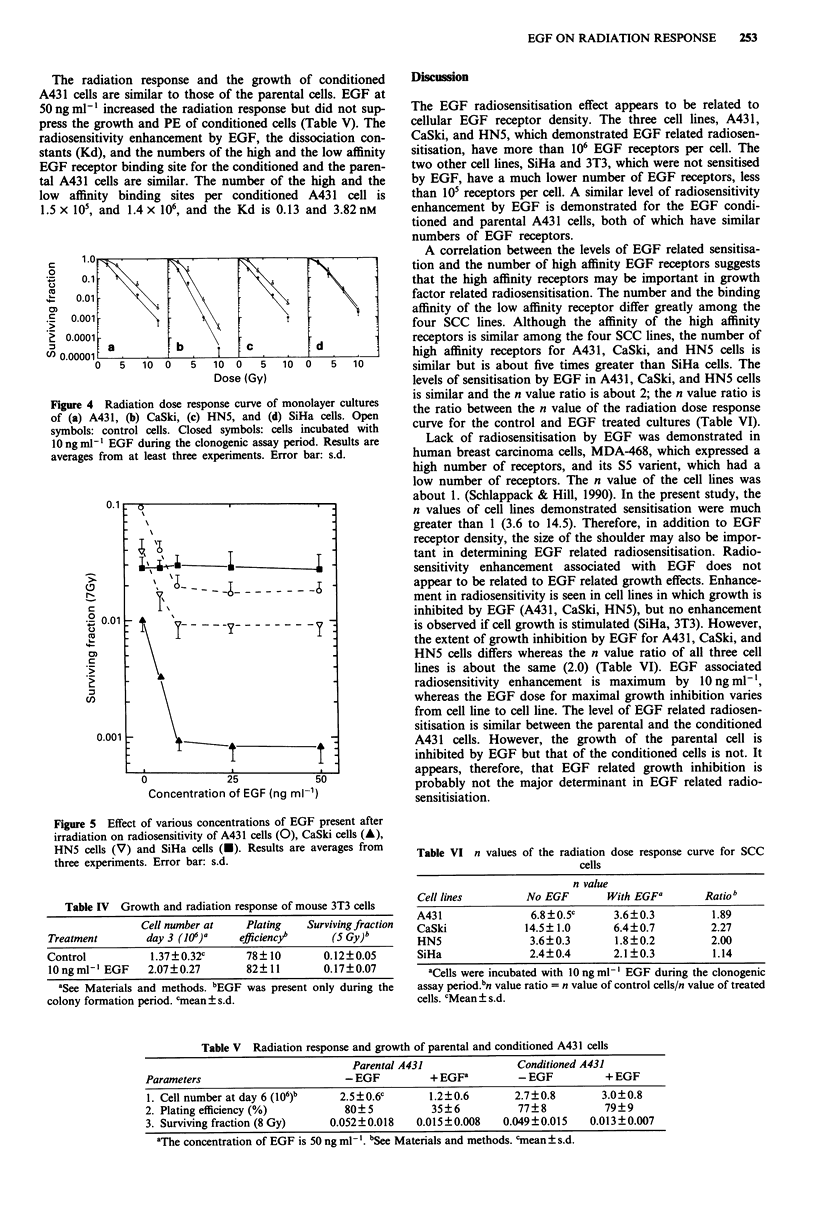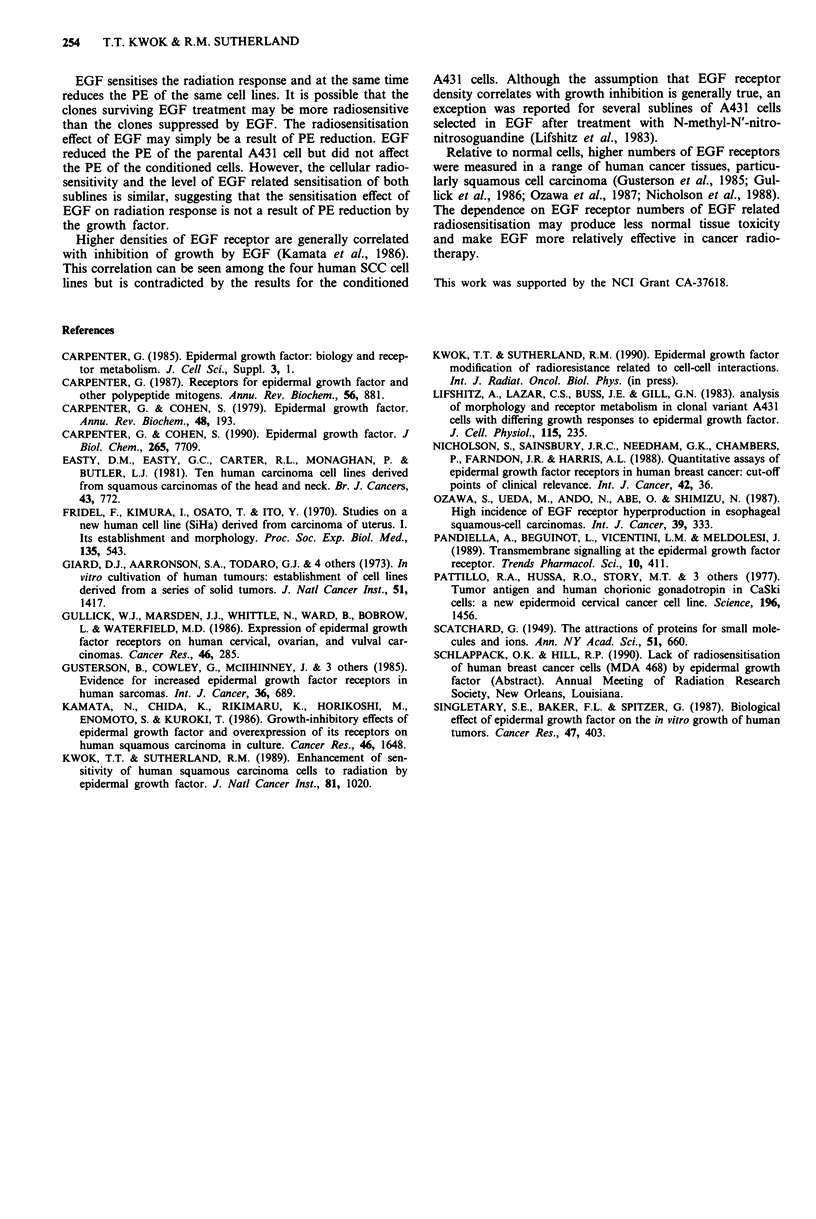# Differences in EGF related radiosensitisation of human squamous carcinoma cells with high and low numbers of EGF receptors.

**DOI:** 10.1038/bjc.1991.286

**Published:** 1991-08

**Authors:** T. T. Kwok, R. M. Sutherland

**Affiliations:** Laboratory of Cell and Molecular Biology, Life Sciences Division, SRI International, Menlo Park, California 94025.

## Abstract

Previous studies have shown that the presence of epidermal growth factor (EGF) after irradiation enhanced the radiosensitivity of CaSki cells. To examine the role of EGF receptor density and related growth response in EGF associated radiosensitisation, four human squamous carcinoma cell lines were used. The total number of EGF receptors for HN5, A431, CaSki, and SiHa cells is 5.2 x 10(6), 1.6 x 10(6), 7.9 x 10(5) and 1.1 x 10(5) respectively, and the dissociation constant (Kd) for low affinity EGF receptors is 11.8, 3.8, 1.7 and 0.8 nM respectively. The Kd for high affinity receptors differs slightly among the four cell lines, 0.09 to 0.21 nM. EGF inhibited the growth of A431, CaSki, and HN5 cells, but stimulated the growth of SiHa cells. Due to the presence of 10 ng ml-1 EGF after irradiation, radiosensitivity enhancement associated with reduced shoulder size of the survival curve was observed. The extent of sensitisation was similar for A431, CaSki, and HN5 cells, with no effect on SiHa cells. At this concentration, EGF present during the clonogenic assay period after irradiation also reduced the plating efficiency (PE) of the monolayer cultures of A431, CaSki, and HN5 cells, but increased that of SiHa cells. The radiation response of mouse 3T3 cells (less than 5,000 receptors) was not sensitised by EGF. A similar level of radiosensitivity enhancement by EGF was observed for parental and conditioned A431 cultures. The conditioned cells were grown in 50 ng ml-1 EGF for 10 weeks and did not demonstrate growth inhibition and PE reduction by treatment with EGF. The EGF receptor numbers and binding affinity of these cells were the same as for the parental cells. The results from the conditioned cells support the hypothesis that EGF related radiosensitisation is EGF receptor density dependent.


*Br. J. Cancer* (1991), **64**, 251–254 © Macmillan Press Ltd., 1991

### Differences in EGF related radiosensitisation of human squamous carcinoma cells with high and low numbers of EGF receptors

T.T. Kwok & R.M. Sutherland

*Laboratory of Cell and Molecular Biology, Life Sciences Division, SRI International, Menlo Park, California 94025, USA.*

**Summary** Previous studies have shown that the presence of epidermal growth factor (EGF) after irradiation enhanced the radiosensitivity of CaSki cells. To examine the role of EGF receptor density and related growth response in EGF associated radiosensitisation, four human squamous carcinoma cell lines were used. The total number of EGF receptors for HN5, A431, CaSki, and SiHa cells is $5.2 \times 10^6$, $1.6 \times 10^6$, $7.9 \times 10^5$ and $1.1 \times 10^5$ respectively, and the dissociation constant (Kd) for low affinity EGF receptors is 11.8, 3.8, 1.7 and 0.8 nM respectively. The Kd for high affinity receptors differs slightly among the four cell lines, 0.09 to 0.21 nM. EGF inhibited the growth of A431, CaSki, and HN5 cells, but stimulated the growth of SiHa cells. Due to the presence of 10 ng $ml^{-1}$ EGF after irradiation, radiosensitivity enhancement associated with reduced shoulder size of the survival curve was observed. The extent of sensitisation was similar for A431, CaSki, and HN5 cells, with no effect on SiHa cells. At this concentration, EGF present during the clonogenic assay period after irradiation also reduced the plating efficiency (PE) of the monolayer cultures of A431, CaSki, and HN5 cells, but increased that of SiHa cells.

The radiation response of mouse 3T3 cells (<5,000 receptors) was not sensitised by EGF. A similar level of radiosensitivity enhancement by EGF was observed for parental and conditioned A431 cultures. The conditioned cells were grown in 50 ng $ml^{-1}$ EGF for 10 weeks and did not demonstrate growth inhibition and PE reduction by treatment with EGF. The EGF receptor numbers and binding affinity of these cells were the same as for the parental cells.

The results from the conditioned cells support the hypothesis that EGF related radiosensitisation is EGF receptor density dependent.

Epidermal growth factor (EGF) induces a variety of changes in cellular metabolism ranging from cell growth to macromolecular synthesis (Carpenter & Cohen, 1979; Carpenter, 1985). The signal transduction process involves binding to a specific transmembrane receptor, internalisation of the receptor complex, activation of various kinds of kinases, changes in phospho-inositol and calcium levels, and activation of c-*myc* and c-*fos* oncogene (Carpenter, 1987; Pandiella *et al.*, 1989; Carpenter & Cohen, 1990). Although EGF did not modify the response of a number of cell lines to anticancer agents such as Adriamycin (Singletary *et al.*, 1987), our previous reports indicated that EGF present after irradiation enhanced the radiosensitivity and modified the radioresistance related to cell-cell interactions of a human squamous cell carcinoma (SCC) cell line, CaSki (Kwok & Sutherland, 1989, 1990). Furthermore, radiosensitisation was also observed when using an EGF-like growth factor, transforming growth factor α (Kwok & Sutherland, unpublished results). In an effort to understand how EGF enhances radiosensitivity, we examined the influence of EGF on the radiation response of four human SCC cell lines demonstrating different growth responses to EGF and different EGF receptor densities.

### Materials and methods

### *Human SCC cells*

Four human SCC cell lines were used. The culture conditions for these cell lines are listed in Table I. For the radiation response experiment, $5 \times 10^5$ cells were seeded in a 60 mm Petri dish. After 2 days, cells in exponential growth phase were irradiated on ice with gamma rays from a Cesium-137 source at 5.0 Gy $min^{-1}$. The cell survival was then measured by a clonogenic assay in which colonies were grown on the plastic surface of a 60 mm Petri dish (Kwok & Sutherland, 1989). Between 200 and 10,000 cells were plated in each dish; the number of cells plated appeared not to alter the EGF effect on radiosensitivity of the four cell lines. At the beginning of a clonogenic assay, various amounts of murine EGF (culture grade, Collaborative Research, Inc., Bedford, MA) were added. At the end of incubation, colonies of more than 50 cells were scored.

For growth studies, $5 \times 10^4$ cells in 5 ml of medium were seeded in a 60 mm Petri dish. At the same time, various amounts of EGF, ranging from 1 to 50 ng $ml^{-1}$, were added. Medium, if applicable, together with EGF, was changed daily beginning on day 3. The total number of cells at day 6 was counted on a hemocytometer.

### *EGF conditioned A431 cells*

The EGF conditioned cells were developed by growing A431 cells in 50 ng $ml^{-1}$ EGF continuously for 10 weeks. Cells were subcultured once a week during this 10 week period. To set up a radiation response experiment, $5 \times 10^5$ cells were grown for 2 days without EGF. After irradiation, the cells were trypsinised and assayed for clonogenic surviving fraction in the presence or absence of 50 ng $ml^{-1}$ EGF. Prior to the measurement of cell growth and EGF receptor density, conditioned cells were grown in medium without EGF for 2 days.

### *Mouse 3T3 cells*

Cells were grown in basal minimum essential medium supplemented with 10% FBS. For radiation response studies, $10^5$

**Table I** Culture condition and origin of human squamous carcinoma cell lines

| *Cell lines*[a] | *Tumour origin* | *Culture medium* | *Duration of clonogenic assay (days)* |
|---|---|---|---|
| A431 | Vulva | DMEM[b] + 20% FBS[c] | 12 |
| CaSki | Cervix | RPMI 1640 + 10% FBS | 16 |
| HN5 | Tongue | DMEM + 10% FBS | 16 |
| SiHa | Cervix | BMEM[d] + 10% FBS | 21 |

[a]The reference for A431 cells is Giard *et al.*, 1973, for CaSki is Pattillo *et al.*, 1977, for HN5 is Easty *et al.*, 1981, for SiHa is Fridel *et al.*, 1970. [b]Dulbecco's minimal essential medium. [c]Foetal bovine serum. [d]Basal minimal essential medium.

Correspondence: T.T. Kwok.
Received 5 November 1990; and in revised form 11 March 1991.

cells were grown in a 60 mm Petri dish for 2 days. After irradiation, cell survival was measured by clonogenic assay; the colony formation period was 10 days; EGF at a final concentration of $10\ \mathrm{ng\ ml^{-1}}$ was added at the beginning of this period. For growth studies, $5 \times 10^4$ cells in 5 ml of medium were seeded in a 60 mm Petri dish with or without $10\ \mathrm{ng\ ml^{-1}}$ EGF. The number of cells in each dish at day 3 was counted on a hemocytometer.

### *EGF receptor binding assay*

For EGF receptor binding studies, $2 \times 10^5$ cells in 2 ml of medium were plated into the wells of a 6-well plate and incubated for 2 days. Cells were then rinsed twice with serum free medium supplemented with 0.1% bovine serum albumin (BSA). Then the cells were incubated with $I^{125}$ human EGF (Amersham Co., Arlington Heights, IL) for 4 h at 4°C. After rinsing with BSA supplemented medium three times, the cells were lysed in 0.5% SDS in 1 M NaOH. The radioactivity was counted in a gamma counter. Nonspecific binding was assessed in the presence of 1 mM cold EGF. The data were analysed by Scatchard plot (Scatchard, 1949).

### Results

Epidermal growth factor significantly inhibited the growth of HN5 cells, slightly inhibited the growth of A431 and CaSki cells, and stimulated the growth of SiHa cells (Figures 1 and 2). The PE of A431, CaSki, and HN5 cells was reduced by almost 50% in the present of $10\ \mathrm{ng\ ml^{-1}}$ EGF; however, a 30% increase in PE was seen for SiHa cells (Table II). The Scatchard plots of EGF receptor for all four cell lines were biphasic, suggesting that there were two types of receptor binding sites, i.e. high and low affinity binding sites (Figure 3). The dissociation constant (Kd) for low affinity receptors of HN5, A431, CaSki, and SiHa cells is 11.80, 3.76, 1.69 and 0.75 nM respectively, and for high affinity receptors is 0.21, 0.13, 0.19 and 0.09 nM respectively. The total number of EGF receptors for HN5 cells is $5.2 \times 10^6$, for A431 cells is $1.6 \times 10^6$, for CaSki cells is $7.9 \times 10^5$, and for SiHa cells is $1.1 \times 10^5$ (Table III).

The radiation dose response curves for these four cell lines are shown in Figure 4. The presence of $10\ \mathrm{ng\ ml^{-1}}$ EGF after irradiation enhanced radiosensitivity, associated with reduced shoulder region of the cell survival curves, of A431, CaSki, and HN5 cells, and there was no change for SiHa cells. However, EGF present only for 48 h before and during irradiation did not have any effect on the radiosensitivity of cells (Kwok & Sutherland, unpublished results). The maximum EGF effect on radiosensitivity enhancement was achieved by $10\ \mathrm{ng\ ml^{-1}}$ (Figure 5). Mouse 3T3 cells that had a low number of EGF receptors (<5,000 per cell) were also examined. EGF at $10\ \mathrm{ng\ ml^{-1}}$ stimulated the growth but did not affect the PE and radiation response of this cell line (Table IV).

**Figure 2** Growth curve of (**a**) A431, (**b**) CaSki, (**c**) HN5, and (**d**) SiHa cells. Open symbols: control cells. Closed symbols: cells incubated with $50\ \mathrm{ng\ ml^{-1}}$ EGF.

**Table II** Plating efficiency of different cell lines

| *Cell lines* | *Plating efficiency (%)* *No EGF* | *With 10 ng ml⁻¹ EGF* |
|---|---|---|
| A431 | 80±5[a] | 35±6 |
| CaSki | 75±7 | 35±5 |
| HN5 | 54±7 | 30±4 |
| SiHa | 70±6 | 92±7 |

[a]Mean ± s.d.

**Figure 1** Effect of various concentrations of EGF on growth of A431 cells (O), CaSki cells (▲), HN5 cells (∇) and SiHa cells (■). Cell numbers at day 6 under different conditions are shown. Results are averages from three experiments. Error bar: s.d.

**Figure 3** Scatchard plot of EGF receptor binding for A431 (O), CaSki (▲), HN5 (∇) and SiHa (■).

**Table III** Affinity and number of EGF receptors in different cell lines

| *Cell lines* | *High affinity receptor* *Kd (nM)* | *Number (10⁵/cell)* | *Low affinity receptor* *Kd (nM)* | *Number (10⁵/cell)* | *Total number (10⁵/cell)* |
|---|---|---|---|---|---|
| HN5 | 0.21±0.03[a] | 1.85±0.03 | 11.80±0.75 | 50.10±0.70 | 51.95±0.76 |
| A431 | 0.13±0.02 | 1.34±0.05 | 3.76±0.67 | 14.20±1.40 | 15.54±1.50 |
| CaSki | 0.19±0.06 | 1.54±0.40 | 1.69±1.50 | 6.31±1.50 | 7.85±1.55 |
| SiHa | 0.09±0.01 | 0.33±0.04 | 0.75±0.25 | 0.81±0.70 | 1.14±0.70 |

[a]mean ± s.d.

The radiation response and the growth of conditioned A431 cells are similar to those of the parental cells. EGF at 50 ng ml$^{-1}$ increased the radiation response but did not suppress the growth and PE of conditioned cells (Table V). The radiosensitivity enhancement by EGF, the dissociation constants (Kd), and the numbers of the high and the low affinity EGF receptor binding site for the conditioned and the parental A431 cells are similar. The number of the high and the low affinity binding sites per conditioned A431 cell is $1.5 \times 10^5$, and $1.4 \times 10^6$, and the Kd is 0.13 and 3.82 nM

**Figure 4** Radiation dose response curve of monolayer cultures of (**a**) A431, (**b**) CaSki, (**c**) HN5, and (**d**) SiHa cells. Open symbols: control cells. Closed symbols: cells incubated with 10 ng ml$^{-1}$ EGF during the clonogenic assay period. Results are averages from at least three experiments. Error bar: s.d.

**Figure 5** Effect of various concentrations of EGF present after irradiation on radiosensitivity of A431 cells (○), CaSki cells (▲), HN5 cells (∇) and SiHa cells (■). Results are averages from three experiments. Error bar: s.d.

**Table IV** Growth and radiation response of mouse 3T3 cells

| *Treatment* | *Cell number at day 3 ($10^6$)*[a] | *Plating efficiency*[b] | *Surviving fraction (5 Gy)*[b] |
|---|---|---|---|
| Control | 1.37±0.32[c] | 78±10 | 0.12±0.05 |
| 10 ng ml$^{-1}$ EGF | 2.07±0.27 | 82±11 | 0.17±0.07 |

[a]See Materials and methods. [b]EGF was present only during the colony formation period. [c]mean ± s.d.

### Discussion

The EGF radiosensitisation effect appears to be related to cellular EGF receptor density. The three cell lines, A431, CaSki, and HN5, which demonstrated EGF related radiosensitisation, have more than $10^6$ EGF receptors per cell. The two other cell lines, SiHa and 3T3, which were not sensitised by EGF, have a much lower number of EGF receptors, less than $10^5$ receptors per cell. A similar level of radiosensitivity enhancement by EGF is demonstrated for the EGF conditioned and parental A431 cells, both of which have similar numbers of EGF receptors.

A correlation between the levels of EGF related sensitisation and the number of high affinity EGF receptors suggests that the high affinity receptors may be important in growth factor related radiosensitisation. The number and the binding affinity of the low affinity receptor differ greatly among the four SCC lines. Although the affinity of the high affinity receptors is similar among the four SCC lines, the number of high affinity receptors for A431, CaSki, and HN5 cells is similar but is about five times greater than SiHa cells. The levels of sensitisation by EGF in A431, CaSki, and HN5 cells is similar and the *n* value ratio is about 2; the *n* value ratio is the ratio between the *n* value of the radiation dose response curve for the control and EGF treated cultures (Table VI).

Lack of radiosensitisation by EGF was demonstrated in human breast carcinoma cells, MDA-468, which expressed a high number of receptors, and its S5 varient, which had a low number of receptors. The *n* value of the cell lines was about 1. (Schlappack & Hill, 1990). In the present study, the *n* values of cell lines demonstrated sensitisation were much greater than 1 (3.6 to 14.5). Therefore, in addition to EGF receptor density, the size of the shoulder may also be important in determining EGF related radiosensitisation. Radiosensitivity enhancement associated with EGF does not appear to be related to EGF related growth effects. Enhancement in radiosensitivity is seen in cell lines in which growth is inhibited by EGF (A431, CaSki, HN5), but no enhancement is observed if cell growth is stimulated (SiHa, 3T3). However, the extent of growth inhibition by EGF for A431, CaSki, and HN5 cells differs whereas the *n* value ratio of all three cell lines is about the same (2.0) (Table VI). EGF associated radiosensitivity enhancement is maximum by 10 ng ml$^{-1}$, whereas the EGF dose for maximal growth inhibition varies from cell line to cell line. The level of EGF related radiosensitisation is similar between the parental and the conditioned A431 cells. However, the growth of the parental cell is inhibited by EGF but that of the conditioned cells is not. It appears, therefore, that EGF related growth inhibition is probably not the major determinant in EGF related radiosensitisiation.

**Table VI** *n* values of the radiation dose response curve for SCC cells

| *Cell lines* | *n value* No EGF | *n value* With EGF[a] | *Ratio*[b] |
|---|---|---|---|
| A431 | 6.8±0.5[c] | 3.6±0.3 | 1.89 |
| CaSki | 14.5±1.0 | 6.4±0.7 | 2.27 |
| HN5 | 3.6±0.3 | 1.8±0.2 | 2.00 |
| SiHa | 2.4±0.4 | 2.1±0.3 | 1.14 |

[a]Cells were incubated with 10 ng ml$^{-1}$ EGF during the clonogenic assay period.[b]*n* value ratio = *n* value of control cells/*n* value of treated cells. [c]Mean ± s.d.

**Table V** Radiation response and growth of parental and conditioned A431 cells

| *Parameters* | *Parental A431* – EGF | *Parental A431* + EGF[a] | *Conditioned A431* – EGF | *Conditioned A431* + EGF |
|---|---|---|---|---|
| 1. Cell number at day 6 ($10^6$)[b] | 2.5±0.6[c] | 1.2±0.6 | 2.7±0.8 | 3.0±0.8 |
| 2. Plating efficiency (%) | 80±5 | 35±6 | 77±8 | 79±9 |
| 3. Surviving fraction (8 Gy) | 0.052±0.018 | 0.015±0.008 | 0.049±0.015 | 0.013±0.007 |

[a]The concentration of EGF is 50 ng ml$^{-1}$. [b]See Materials and methods. [c]mean ± s.d.

EGF sensitises the radiation response and at the same time reduces the PE of the same cell lines. It is possible that the clones surviving EGF treatment may be more radiosensitive than the clones suppressed by EGF. The radiosensitisation effect of EGF may simply be a result of PE reduction. EGF reduced the PE of the parental A431 cell but did not affect the PE of the conditioned cells. However, the cellular radiosensitivity and the level of EGF related sensitisation of both sublines is similar, suggesting that the sensitisation effect of EGF on radiation response is not a result of PE reduction by the growth factor.

Higher densities of EGF receptor are generally correlated with inhibition of growth by EGF (Kamata *et al.*, 1986). This correlation can be seen among the four human SCC cell lines but is contradicted by the results for the conditioned A431 cells. Although the assumption that EGF receptor density correlates with growth inhibition is generally true, an exception was reported for several sublines of A431 cells selected in EGF after treatment with N-methyl-N′-nitro-nitrosoguandine (Lifshitz *et al.*, 1983).

Relative to normal cells, higher numbers of EGF receptors were measured in a range of human cancer tissues, particularly squamous cell carcinoma (Gusterson *et al.*, 1985; Gullick *et al.*, 1986; Ozawa *et al.*, 1987; Nicholson *et al.*, 1988). The dependence on EGF receptor numbers of EGF related radiosensitisation may produce less normal tissue toxicity and make EGF more relatively effective in cancer radiotherapy.

This work was supported by the NCI Grant CA-37618.

### References

CARPENTER, G. (1985). Epidermal growth factor: biology and receptor metabolism. *J. Cell Sci.*, Suppl. **3**, 1.

CARPENTER, G. (1987). Receptors for epidermal growth factor and other polypeptide mitogens. *Annu. Rev. Biochem.*, **56**, 881.

CARPENTER, G. & COHEN, S. (1979). Epidermal growth factor. *Annu. Rev. Biochem.*, **48**, 193.

CARPENTER, G. & COHEN, S. (1990). Epidermal growth factor. *J Biol. Chem.*, **265**, 7709.

EASTY, D.M., EASTY, G.C., CARTER, R.L., MONAGHAN, P. & BUTLER, L.J. (1981). Ten human carcinoma cell lines derived from squamous carcinomas of the head and neck. *Br. J. Cancers*, **43**, 772.

FRIDEL, F., KIMURA, I., OSATO, T. & ITO, Y. (1970). Studies on a new human cell line (SiHa) derived from carcinoma of uterus. I. Its establishment and morphology. *Proc. Soc. Exp. Biol. Med.*, **135**, 543.

GIARD, D.J., AARRONSON, S.A., TODARO, G.J. & 4 others (1973). *In vitro* cultivation of human tumours: establishment of cell lines derived from a series of solid tumors. *J. Natl Cancer Inst.*, **51**, 1417.

GULLICK, W.J., MARSDEN, J.J., WHITTLE, N., WARD, B., BOBROW, L. & WATERFIELD, M.D. (1986). Expression of epidermal growth factor receptors on human cervical, ovarian, and vulval carcinomas. *Cancer Res.*, **46**, 285.

GUSTERSON, B., COWLEY, G., MCIIHINNEY, J. & 3 others (1985). Evidence for increased epidermal growth factor receptors in human sarcomas. *Int. J. Cancer*, **36**, 689.

KAMATA, N., CHIDA, K., RIKIMARU, K., HORIKOSHI, M., ENOMOTO, S. & KUROKI, T. (1986). Growth-inhibitory effects of epidermal growth factor and overexpression of its receptors on human squamous carcinoma in culture. *Cancer Res.*, **46**, 1648.

KWOK, T.T. & SUTHERLAND, R.M. (1989). Enhancement of sensitivity of human squamous carcinoma cells to radiation by epidermal growth factor. *J. Natl Cancer Inst.*, **81**, 1020.

KWOK, T.T. & SUTHERLAND, R.M. (1990). Epidermal growth factor modification of radioresistance related to cell-cell interactions. *Int. J. Radiat. Oncol. Biol. Phys.* (in press).

LIFSHITZ, A., LAZAR, C.S., BUSS, J.E. & GILL, G.N. (1983). analysis of morphology and receptor metabolism in clonal variant A431 cells with differing growth responses to epidermal growth factor. *J. Cell. Physiol.*, **115**, 235.

NICHOLSON, S., SAINSBURY, J.R.C., NEEDHAM, G.K., CHAMBERS, P., FARNDON, J.R. & HARRIS, A.L. (1988). Quantitative assays of epidermal growth factor receptors in human breast cancer: cut-off points of clinical relevance. *Int. J. Cancer*, **42**, 36.

OZAWA, S., UEDA, M., ANDO, N., ABE, O. & SHIMIZU, N. (1987). High incidence of EGF receptor hyperproduction in esophageal squamous-cell carcinomas. *Int. J. Cancer*, **39**, 333.

PANDIELLA, A., BEGUINOT, L., VICENTINI, L.M. & MELDOLESI, J. (1989). Transmembrane signalling at the epidermal growth factor receptor. *Trends Pharmacol. Sci.*, **10**, 411.

PATTILLO, R.A., HUSSA, R.O., STORY, M.T. & 3 others (1977). Tumor antigen and human chorionic gonadotropin in CaSki cells: a new epidermoid cervical cancer cell line. *Science*, **196**, 1456.

SCATCHARD, G. (1949). The attractions of proteins for small molecules and ions. *Ann. NY Acad. Sci.*, **51**, 660.

SCHLAPPACK, O.K. & HILL, R.P. (1990). Lack of radiosensitisation of human breast cancer cells (MDA 468) by epidermal growth factor (Abstract). Annual Meeting of Radiation Research Society, New Orleans, Louisiana.

SINGLETARY, S.E., BAKER, F.L. & SPITZER, G. (1987). Biological effect of epidermal growth factor on the *in vitro* growth of human tumors. *Cancer Res.*, **47**, 403.